# Review of Traditional Chinese Medicines for Common Complications Related to Hemodialysis: An Evidence-Based Perspective

**DOI:** 10.1155/2021/9953986

**Published:** 2021-07-13

**Authors:** Yan-Wen Liu, Yung-Tang Hsu, Wen-Chin Lee, Ming-Yen Tsai

**Affiliations:** ^1^Department of Chinese Medicine, Kaohsiung Chang Gung Memorial Hospital and Chang Gung University College of Medicine, Kaohsiung 83301, Taiwan; ^2^Division of Nephrology, Department of Internal Medicine, Kaohsiung Chang Gung Memorial Hospital and Chang Gung University College of Medicine, Kaohsiung 83301, Taiwan

## Abstract

Renal replacement therapy is an important therapy for prolonging life in end-stage renal disease (ESRD) populations, and, in Taiwan, hemodialysis (HD) is the choice for most patients with ESRD. Although HD is effective for prolonging life, it is sometimes associated with complications that patients and doctors have to cope with every day, such as intradialytic hypotension, dialysis disequilibrium syndrome, and muscle cramps. Traditional Chinese medicine (TCM) is a complementary and alternative therapy that has been recognized for its efficacy in treating a variety of diseases by the World Health Organization. Nowadays, the clinical practice of TCM for HD-related complications has received attention for its effectiveness and safety. In this article, we summarize the TCM viewpoint and different TCM interventions for HD-related complications, such as Chinese herbal medicine, acupuncture, herbal acupoint therapy, auricular acupoints, and moxibustion. In the ESRD population, TCM is able to balance Yin and Yang, prevent cardiovascular accidents, control blood pressure, and relieve pain. More importantly, TCM may also improve common HD-related complications such as uremic symptoms, imbalance of electrolyte and fluid status, insomnia, and malnutrition. The mechanism of TCM is considered related to the regulation of autonomous functions and the activation of biologically active chemical substances. According to the studies noted in this review article, TCM has been proven effective for HD-related complications. However, more well-designed and rigorous research will be necessary to reveal the underlying mechanisms in the future.

## 1. Introduction

As the average life expectancy increases, the uremic population increases. According to a report from the United States Renal Data System (USRDS), in 2008, the prevalence of end-stage renal disease (ESRD) in the Taiwanese population was 2,311 per million, which was the highest worldwide [1]. Despite their progression to ESRD, these patients could still prolong their lives by renal replacement therapy, which includes hemodialysis (HD), peritoneal dialysis (PD), and kidney transplantation. In Taiwan, thanks to the National Health Insurance (NHI) program, convenience of medical care, and advanced development of medical technologies, ESRD patients tend to choose HD. In 2015, a total of 11,179 new cases needing dialysis were diagnosed, and, of these, 9,988 received HD treatment, while 1,191 were treated with PD [2].

Although HD can effectively relieve uremic symptoms and improve the quality of life (QoL), it inevitability entails complications. Common complications related to HD include intradialytic hypotension (IDH), muscle cramps, nausea and vomiting, headache, chest pain, and back pain, of which the first three are the most common [3]. Some of the abovementioned complaints can also be categorized as dialysis disequilibrium syndrome (DDS). Some causes are iatrogenic, whereas others might be related to idiosyncratic reactions [4]. By ultrafiltration (UF), HD removes excess metabolic substances (i.e., uremia, creatinine, phosphorus, and potassium) and water from the blood, and it also replenishes the body's calcium and bicarbonate to purify blood and to maintain electrolysis and acid-base balance. During each HD treatment, blood is drawn from the patient, passes through an artificial kidney, and then returns to the patient's body at a rate of 200–300 milliliters per minute. Each HD treatment lasts about 4 hours, and treatments are performed 2 to 3 times per week. The change in the blood volume during the whole HD procedure, in the view of traditional Chinese medicine (TCM), is similar to artificially induced deficiencies of both liquid and blood and consumes health essence energy (also called essence-*Qi*). Although such treatments do not cause disorientation of both *Qi* and blood, the dramatic change in fluid status and electrolyte imbalance during HD are thought to result in temporary deficiencies of both Yin and Yang.

TCM is widely used in Asia and has been found to be effective in the treatment of chronic kidney disease (CKD) [5]. Its theoretical system, which is clearly different from that of Western medicine (WM), is based on the harmony of Yin-Yang and *Qi*-blood. The choices of TCM interventions and their prescriptions are determined according to each patient's constitution and disease progression. However, many nephrologists discourage the concurrent use of TCM due to the potential for adverse interactions between Chinese and Western drugs, which may lead to electrolyte imbalance. In the past, numerous reports of kidney injury caused by TCM herbs containing aristolochic acids and by folk remedies made their safety more suspicious [6, 7]. An early questionnaire-based study revealed that 10% of patients with CKD had privately used TCM, and the ESRD risk increased by 20% among TCM users [8].

To maintain quality control in manufacturing, the processing, packaging, and repackaging of the raw materials of Chinese herbal medicine (CHM) have been governed by a central competent health authority since 2006 [9]. All aristolochic-acid-containing herbs are also strictly prohibited in Taiwan. Most retrospective cohort studies based on NHI data have supported the potential advantages of CHM use by patients with CKD, despite the increased risk of chronic kidney disease among users of nonprescribed Chinese herbal medicine in Taiwan [10–12], particularly in improving QoL, preventing CKD progression, and reducing ESRD risk. Moreover, CHM could lower the risk of CKD if patients follow the syndrome-based prescriptions by TCM doctors [12]. However, few studies have demonstrated that TCM can mitigate HD-related complications. Therefore, this article discusses the pathophysiology and mechanisms of these complications from the perspectives of both WM and TCM, and it also retrospectively reviews the role of TCM as a complementary and alternative therapy for these common HD-related complications.

## 2. Intradialytic Hypotension

Intradialytic hypotension (IDH) is the most common complication, with an incidence of 25–55% in HD patients [13]. According to a previous study, 75% of HD patients had at least one episode of IDH [14]. The National Kidney Foundation's Kidney Disease Outcomes Quality Initiative defines IDH as a decrease in systolic blood pressure (BP) of ≥20 mmHg or a decrease in mean arterial pressure of ≥10 mmHg, accompanied by symptoms including lightheadedness, muscle cramping, nausea and vomiting, dyspnea, and abdominal pain [15]. The common causes of IDH can be roughly summarized as rapid or excessive UF, a rapid change in plasma osmolality, wrong target weight, dysautonomia, abnormal cardiac function, infection, prescription of antihypertensives, adverse reaction toward dialysate, and ingestion of a meal immediately before or during HD [15]. The acute management of IDH includes decreasing the rate of or stopping UF, administration of oxygen, positioning in the Trendelenburg position, and fluid supplementation if necessary [15]. If hypotension persists after the above interventions, it is important to evaluate possible serious causes. For prevention in patients with recurrent episodes, a stepped approach and evaluation are recommended. The first-line approach includes reassessing the target weight, avoiding meals before or during dialysis, withholding antihypertensives before HD, and limiting sodium intake. The second-line approach includes evaluation of cardiac function, the use of a cool dialysate, and an increase in dialysis time and/or frequency. The third-line approach includes administration of midodrine and/or shifting to other forms of HD [15]. Patients with higher incidence of IDH usually have higher morbidity and mortality [16]. IDH can lead to early cessation of the HD process and resultant failure to achieve the target weight. In addition, frequent hypotension can lead to bowel ischemia, stroke, myocardial infarction, or even fistula thrombosis [15]. Therefore, effective prevention of IDH is beneficial for these patients to improve their QoL and reduce their symptomatic disorders and mortality rate.

Although the classical literature in TCM lists no disease named hypotension, the clinical manifestations suggest the categorization of IDH into certain TCM diseases, such as “dizziness syndrome,” “juc collapse,” and “asthenic disease.” As recorded in Jing Yue Quan Shu, “no deficiency, no dizziness” and “Those *Qi* deficiency and suddenly collapsed people must have clinical manifestations with lifelessness, pallor, cold body, and weak pulse. This is so-called *Qi* collapse syndrome.” These statements describe symptoms resembling those of IDH, such as cold limbs, general weakness, shortness of breath, and sweating. Hence, in the view of TCM, most IDH patients have clinical manifestations of deficiency syndrome. For example, Wang et al. analyzed the TCM syndromes of 44 IDH patients and concluded that, among this population, the percentage of *Qi* deficiency syndrome was 88.63%, whereas the percentage of Yang-*Qi* deficiency syndrome was 61.36% [17]. Among this population with *Qi* deficiency syndrome, the majority have *Qi* deficiency of both the spleen and kidney and Yang deficiency of both the spleen and kidney. Moreover, Wang et al. found percentages of mixed deficiency and excess syndrome of up to 95.45%. The excess syndrome consisted of mostly blood stasis, dampness, windiness, and water vapor. According to the results, we can assume that the body constitution of these IDH patients had deficiencies in both the spleen and kidneys. HD removes water, dampness, turbidity, and toxins out of the body, as well as consuming *Qi* and damaging the blood. In other words, HD could lead to a deficiency of Yin, consumption of *Qi*, and expulsion of Yang deficiency, leading to the syndromes of “*Qi* desertion along with fluid depletion” or “*Qi* desertion along with blood depletion.” Liu Zhou and Tang [18] assumed that the syndrome of IDH could mainly be attributed to “deficiency of both *Qi* and Yin” or “*Qi* desertion along with blood depletion.” The early stage of IDH is a syndrome of deficiency of both *Qi* and Yin, which could be treated under the TCM principle of replenishing *Qi* and nourishing Yin. Hence, administration of herbs such as *Rx. Astragali* and Sheng Mai Yin (SMY) for “replenishing *Qi* and recovering pulse” and “tonifying *Qi* and producing fluid” is a feasible choice. On the other hand, the syndrome of the late IDH stage is “*Qi* desertion along with blood depletion,” which could be treated under the principle of “invigorating *Qi* and preventing exhaustion” and be correspondingly managed with Shen Fu injection (SFI). Liu Zhou and Tang also reported the case of a 62-year-old female IDH patient who experienced hypovolemic shock and was resuscitated following the urgent administration of SFI, with no notable side effects [18]. SFI is a proprietary Chinese medicine. According to previous studies, it can effectively improve the microcirculation of shocked rats by increasing the microcirculation of blood in the capillaries [19], improving oxygen metabolism in tissues, and strengthening cardiac output [20]. A particular problem with safety is the potential dose-dependent cardiotoxicity of toxic aconite [21, 22]. However, no significant hepatic or renal dysfunction was reported in SFI in the included trials of IDH. The only reported adverse events related to SFI in the literature include headache and skin rashes that lasted only one day [23]. Therefore, medical practitioners should be aware of the potential adverse effects associated with SFI.

Tsai et al. use CHM powder, including *Semen Sinapis*, *Rhizoma Corydalis*, and *Hb. cum Rx. Asari,* as herbal acupoint therapy (HAT) applied to acupoints to stimulate the BP, such as Yongquan (KI1) and Guanyuan (RN4), during HD for 1 month [24]. The HAT group was found to have a significantly lower frequency of IDH episodes, fewer nursing interventions, a lower failure rate of nursing interventions, and earlier discontinuation of HD than those in the sham group. In addition, the recovery time from fatigue after HD was shorter in the HAT group than in the sham group.

Sheng Mai injection (SMI) is produced by using modern methods to extract the active ingredients of SMY, which theoretically act faster and have better efficacy. Studies have also shown that SMI can reduce cardiac mass and left ventricular mass in rats with congestive heart failure, indicating a beneficial effect on ventricular remodeling and cardiac function [25]. SMI use has been viewed as a safe treatment for unstable hemodynamics, except in some cases where headache, nausea, and bradycardia were reported [26]. Guolan analyzed the therapeutic effect of SMY combined with modified Si Miao San decoction on the basis of SMI [27]. After 12 weeks of intervention, compared with the SMI alone group, the decoction group exhibited much more significant improvements in BP, hemoglobin, albumin, and C-reactive protein. The combined use of these three drugs has the synergistic effects of replenishing *Qi*, nourishing Yin, and cleaning heat and dampness; thus, it is beneficial for relieving IDH. However, these explanations of the mechanisms are based on TCM theory, so future scientific studies will be needed to identify the underlying physiological mechanisms.

To sum up, IDH can be viewed as a syndrome of deficiency in origin and excess in superficiality. In other words, it is a complicated TCM syndrome comprising not only renal disease as a basis featured by “prolonged illness usually accompanied with essence *Qi* deficiency” but also excess syndromes such as phlegm, dampness, stasis, and turbidity.

## 3. Intradialytic Hypertension

On the other hand, about 10–15% of HD patients develop intradialytic hypertension [28]. This usually occurs during the second or third hour of HD after significant UF has taken place, when the increase in the BP has been resistant to UF. Therefore, the pathogenesis is still unclear. Some researchers have speculated that it may be related to an imbalance of nitric oxide and endothelin-1 or dysfunction of endothelial cells [29]. The optimal management for intradialytic hypertension remains to be determined at present. Administration of antihypertensive medications, reevaluation of dry weight, and consideration of dialysate with lower sodium concentrations are worth a try [28]. Moreover, some observatory studies have found that intradialytic hypertension is associated with poor prognosis, such as high mortality and hospitalization rates [30, 31].

Intradialytic hypertension can also be categorized as “dizziness syndrome” in TCM theory. Prolonged renal disease means injured “nephron,” which would cause deficiencies in both *Qi* and Yin. Moreover, HD can remove a large amount of water from the body. In TCM theory, blood and water can be seen as having the same origin in the human body. Therefore, HD would increase the severity of conditions such as consumption of fluids and exhaustion of Yin essence, which would lead to undernourishment of the liver, resulting in ascendant hyperactivity of liver-Yang at the same time, which would cause hypertension. In clinical practice, acupoints that nourish Yin by suppressing hyperactive Yang or tonifying the kidney with nourishing Yin, such as Sanyinjiao (SP6), Taixi (KI3), and Shenguan (LE44), are possible treatments [32]. The TCM treatment principle of returning fire to the origin could also be taken into consideration. This means storing the rising Yang-*Qi* in the lower jiao to balance Yin and Yang and harmonize the *Qi* and blood. On the basis of the above theory, Li compared the effects of auricular acupressure plus HAT with routine care for intradialytic hypertension [33]. The administration of auricular acupoints based on TCM syndrome differentiation was as follows. For the syndrome of ascendant hyperactivity of liver-Yang, the heart of the posterior surface (P1), apex (EX-HN6), liver (CO12), and superior triangular fossa (TF1) were chosen; for the syndrome of deficiency of both Yin and Yang, the spleen (CO13), kidney (CO10), spleen of the posterior surface (P3), and groove of the posterior surface (GPS) were chosen; for the syndrome of phlegm-dampness blocking the middle-jiao, triple energy (CO17), spleen (CO13), spleen of the posterior surface (P3), groove of the posterior surface (GPS), and sympathetic (AH6a) were chosen. As for HAT, a herbal powder including *Ligusticum striatum, Achyranthes bidentate*, and *Tetradium ruticarpum* was applied over Yongquan (KI1). After 1 month of intervention, significant improvements were noted in BP, frequency of HD-related complications, and QoL in the auricular acupressure plus HAT group.

For ESRD patients, BP should be closely monitored during HD. Clinical practice and some experiments have proven that acupuncture is effective for BP [34]. However, research on TCM treatment for intradialytic hypertension is still insufficient. Further large-scale and rigorous research is necessary.

## 4. Muscle Cramps

Muscle cramps, or spasms, are the second most common HD complication, with an incidence of 5–20% [13]. Older patients and those with longer HD duration are at higher risk for muscle cramps. When this complication presents, it usually manifests as acute tonic contraction and severe pain with durations of seconds to minutes. In such cases, the HD is terminated early due to severe pain and discomfort [35]. Muscles in the limbs and abdomen might be affected; however, those in the lower limbs are more susceptible, followed by those in the abdomen and upper limbs [36]. Muscle cramps usually occur near the end of HD treatment sessions, so they are considered to be related to extracellular fluid or blood osmolality [35]. Currently, the causes of intradialytic muscle cramps are attributed to insufficient blood volume, hyponatremia, tissue hypoxia, hypomagnesemia, carnitine deficiency, and elevated leptin [36]. For symptom relief, hypertonic fluid supplements, discontinuation of UF, or decreasing the HD blood flow rate is employed, whereas, for prevention, strict body weight control, prevention of intradialytic hypotension, supplementation with carnitine, or even vitamin E can be considered [37].

Muscle cramps were called “Jin Bi” or “Bi-syndrome” in ancient China. In Yi Zong Jin Jian, “Jin Bi manifests as cramps, arthralgia, and difficulty of joint extension.” The HD procedure can be seen as causing an inevitable massive loss of body fluid and electrolyte, which in the TCM view means that *Qi* cannot nourish the meridian and the blood cannot support the fascia and tendon, hence eventually resulting in muscle cramps. According to clinical manifestation, Bi-syndrome can be subdivided into “migratory Bi,” “pain Bi,” and “stationary Bi,” so we can choose the treatment based on syndrome differentiation. Moreover, based on TCM principles, such as “the spleen governs the muscle,” “the liver stores blood and governs the tendons,” and “the kidney governs the bones,” muscle cramps can also be treated under the visceral theory of the spleen, liver, and kidney. According to the Bi-theory of the Su Wen, “Pain is mainly due to cold.” Yufang Duan et al. [38] applied moxibustion over Zusanli (ST36), Sanyinjiao (SP6), and Guanyuan (CV4) for patients with muscle cramps and found that, compared to a routine care group, the moxibustion group showed significant improvements in the pain scores, frequency, and duration of muscle cramps. Massage can also be used to manage or relieve symptoms during acute muscle cramping. Jing applied massage over Hegu (LI4), Taichong (LR3), Chengshan (BL57), Chengjin (BL56), Yanglingquan (GB34), and Zusanli (ST36) for muscle cramps, and it was found that massage could significantly relieve symptoms in 5 minutes and 10 minutes, unlike routine care [39]. According to a single-blind clinical study, acupressure over Chengshan (BL57), Shuigou (GV26), Taichong (LR3), Guanyuan (CV4), Qihai (CV6), Yongquan (KI1), Lieque (LU7), and Taiyuan (LU9) 15 mins before HD could reduce the frequency and severity of muscle cramps [40]. In another research from China, Hu et al. suggested that muscle cramps could be mainly attributed to the syndrome of Yin deficiency in both the liver and kidney [41]. Hence, acupuncture over Ququan (LR8), Fuliu (KI7), Guanyuan (CV4), Qimen (LR14), Ganshu (BL18), and Shenshu (Bl23) was chosen correspondingly. After 10 days of intervention once a day for 20 minutes each time, symptoms including tonic contraction and convulsions were controlled and relieved. Modern medical research has found that Shao Yao Gan Cao Tang (SYGCT), a TCM formula, is effective for relief of muscle cramps [42], as recorded in Shang Han Za Bing Lun, which states that “administration of SYGCT could make the patient's legs straight.” In addition, two studies from Japan also found that SYGCT has an inhibitory effect on the contraction of skeletal muscles [43, 44]. SYGCT can immediately relieve muscle cramps in HD patients at home and prevent such episodes; in addition, it can also reduce the duration of muscle cramps and pain scores during HD sessions, as reported by Hyodo et al. [44]. A study by Hinoshita et al. [43] found that administration of SYGCT can prevent muscle cramps in patients undergoing HD and confirmed that it directly blocks stimulated twitching in phrenic nerve-diaphragm preparations in rats. It was found that the potassium level had increased to 5.8 mEq/L at the end of treatment in only one patient. As mentioned in an earlier systematic review of muscle cramps by Ota et al. [45], they also suggest that patients be monitored for pseudoaldosteronism during long-term use of SYGCT.

## 5. Nausea, Vomiting, and Headache

HD-induced nausea and vomiting is the third most common HD complication, with an incidence of 5–15%; however, the incidence of HD-induced headache is lower, with an incidence of about 5% [13]. Although the mechanisms of these complications are still unclear, they could be mainly attributed to longer treatment times and/or inadequate UF. However, based on the clinical manifestations, they could also be categorized as DDS, and, in some cases, psychological factors should be taken into consideration [3]. In most patients, HD-related headaches resolve in 72 hours. However, in some recurrent cases, it is necessary to evaluate metabolic disturbances (such as hypoglycemia, hyponatremia, and hypernatremia), uremia, subdural hematoma, and drug effects [3]. In addition, some literature has indicated that caffeine intake is associated with HD-related headaches [46]. Currently, when DDS is suspected, treatment involves symptom relief and intervention for specific causes.

The TCM syndrome of these HD patients is mostly deficiencies in both the spleen and kidney. Deficiency in the spleen involves a failure in the distribution of fluids, whereas a deficiency in the kidney entails failure to transform *Qi*. In other words, deficiencies of both the spleen and kidney would lead to an inability of *Qi* to rise, turbid fluid being regurgitated instead of down flowing, and eventual obstruction of pathogens, which cause nausea and vomiting. Chen Guimei and Kuang [47] applied *Zingiber officinale* as HAT over Shenque (CV8) plus acupressure massage over Zhongwan (CV12), Neiguan (PC6), and Zusanli (ST36) and found that, compared to metoclopramide treatment, HAT along with acupressure could significantly relieve HD-related nausea and vomiting. Currently, some research has strongly indicated the antiemetic effect of *Zingiber officinale* in both clinical studies and animal experimentation [48, 49]. Another case report from Taiwan has proposed a new viewpoint [50]; namely, uremia-induced vomiting could be managed by syndrome differentiation under the theory of reverting the Yin channel. For instance, the Shang Han Za Bing Lun states that “Dang Gui Si Ni Tang (DGSNT) is administered for cold limbs and weak pulse; if the patient suffers from chronic inner cold, DGSNT plus *Fr. Evodiae* and *Rz. Zingiberis Recens* can be chosen.” Impaired renal function leads to uremia retention in the body; hence, HD patients will complain of nausea and vomiting. In the TCM view, DGSNT plus *Fr. Evodiae* and *Rz. Zingiberis Recens* is capable of dispelling cold to descend adverse-rising *Qi*, warming the liver and harmonizing the stomach, so it could eliminate uremia, correct an acid-base imbalance, and eventually relieve vomiting. *Herba Asari*, a component of DGSNT, is widely used for the treatment of the common cold, headache, toothache, and rheumatic arthralgia in TCM. Inappropriate use of the single herb can lead to acute toxic reactions such as respiratory paralysis, headache, abdominal pain, vomiting, restlessness, and drowsiness. Although *Herba Asari* is in Aristolochiaceae family, the content of aristolochic acid is much lower than other species, and the decoction of the root part of *Herba Asari* is currently recommended for use [22]. *Fr. Evodiae* has been used in China for thousands of years as an analgesic, antiemetic, anti-inflammatory, and antidiarrheal drug. However, it is a toxic drug that causes hepatotoxicity in humans; Cai et al. reported that aqueous extract of *Fr. Evodiae* can produce obvious cumulative hepatotoxicity [51]. Its alkaloid isolates have also been shown to have antiplatelet activity in an *in vivo* study [52], so HD patients must exercise caution when concurrently using aspirin and heparin.

Xie Lanqian [53] suggested that the body constitution of HD-related headache patients could have a pathological basis in liver dysfunction in dispersion; deficiency of *Qi*, blood, and Yin essence; and stasis of blood. HD can aggravate dysregulation of *Qi*-blood and Yin essence, failure of healthy Yang to increase, malnutrition of the meridian, ascendant hyperactivity of Liver-Yang due to deficiency of the liver and kidney, or strangulation of essence-*Qi.* All of the above TCM syndromes would lead to malnutrition of the brain and insufficiency of the meridian, which eventually would cause headache. Based on the above TCM syndrome differentiation, auricular acupoints were chosen for treatment. Seeds of *Vaccaria segetalis* were applied over the following auricular acupoints: shenmen (TF4), subcortex (AT4), occiput (AT3), kidney (CO10), liver (CO12), and spleen (CO13). In addition, self-acupressure was encouraged while at home or during HD. It was found that, compared with a routine treatment group, patients in the acupressure group exhibited significant improvements in the severity of headache, duration of maintenance after acupressure, QoL, fatigue, and depressive mood.

## 6. Chest Pain

The incidence of chest pain is lower than that of the abovementioned complaints, only about 2–5% [13], and chest pain might be attributed to intradialytic hypotension or DDS. However, life-threatening situations such as angina, hemolysis, and air embolism should not be taken lightly [3]. Large-scale studies have established the association between CKD and cardiovascular diseases [54, 55]. Hence, in cases of acute onset of chest pain during HD, angina should be taken into consideration first. Oxygen therapy, antiplatelet agents, and slower UF rates can be administered in cases of acute angina, while morphine and nitroglycerin can be considered as necessary. For prevention of angina, nitrate or *β* blockers prior to HD can be administered; however, vital signs should be closely monitored, for those medications may aggravate IDH [3].

HD-induced chest pain can be categorized as “chest impediment” in TCM. Lu et al. [56] suggested that the body constitution of these patients could be a deficiency of kidney*-Qi* and insufficiency of heart-blood in origin, along with obstruction of turbid phlegm, blood stasis, and pathogenic toxins in superficiality. Deficient *Qi* cannot transport blood, which would lead to strangulation of *Qi* and blood in the heart and obstruction of turbid phlegm and blood stasis in the chest, and it could eventually cause chest tightness and chest pain. In their research, they found that both She Xiang Bao Xin Pill (SXBXP, derived from Su He Xiang Pill) and isosorbide dinitrate could relieve HD-related chest pain and other complications. However, isosorbide dinitrate might not be suitable for chest pain, for it can exaggerate the condition of the ischemic heart due to decreased BP, which would stimulate sympathetic nerves, increase the heart rate, shorten the diastolic period, and reduce perfusion of the coronary artery.

SXBXP contains seven TCM materials: *Moschus*, *Rx. Ginseng*, *Cx. Cinnamomi*, *Calculus bovis*, *Styrax*, *Venenum Bufonis*, and *Borneolum*. In preclinical studies, SXBXP has demonstrated therapeutic effects on cardiovascular diseases via various beneficial mechanisms, including regulating angiogenesis and coronary artery dilation, repressing inflammation and oxidation stress, and protecting vascular endothelium, as well as maintaining BP in clinical practice [57]. A reported toxicity profile showed that coagulation function was altered immediately after oral administration of SXBXP in female rats, which implies that it might be advisable to monitor coagulation indexes and blood lipids in female patients [58]. The side effects of SXBXP mainly include tongue numbness, nausea, emesis, rashes, dizziness, and palpitations. Currently, the evidence on the dosage and safety of SXBXP for treatment of HD-related chest pain remains insufficient because most countries restrict the usage of *Moschus*. The reason is that its ingestion or overdose can cause adverse reactions similar to digoxin, such as abnormal respiratory pattern, heart failure, nausea, dizziness/headache, and even coma or convulsions [59].

## 7. Dialysis Disequilibrium Syndrome

Dialysis disequilibrium syndrome (DDS) is characterized by a range of neurologic symptoms that affect patients during hemodialysis, including hypertension, nausea, vomiting, headache, chest pain, blurred vision, seizures, and conscious disturbances. Most cases are resolved spontaneously, but, in some cases, the condition can worsen to coma or even death [60]. Thanks to advancements in modern medical treatment, the incidence of DDS has gradually decreased [61]. Most DDS symptoms occur after the start of HD, but some, such as muscle cramps and dizziness, may present near the end of HD [60]. DDS mostly affects people receiving HD for the first time or those who have missed multiple consecutive dialysis treatments. Risk factors include first HD treatment, markedly elevated blood urea nitrogen concentration prior to a dialysis session, extremes of age, preexisting neurologic diseases (e.g., head trauma, stroke, and seizure), conditions that could be associated with cerebral edema, and increased permeability of the blood-brain barrier (e.g. sepsis and vasculitis) [60]. At present, the mechanism of DDS can be attributed to cerebral edema caused by rapid osmotic changes due to urea. HD rapidly removes small solutes such as urea from blood. The rapid change in the blood urea content in patients who have marked azotemia acutely and significantly lowers plasma osmolality, hence creating a transient osmotic gradient between plasma and brain cells. This gradient causes a water shift in neurons which produces cerebral edema [62, 63]. For prevention, shortening the duration of HD, lowering the blood flow, modifying the sodium concentration, or restriction of protein intake can be taken into consideration. Treatment to relieve symptoms, such as corresponding management for seizures or nausea/vomiting, should be administered first upon DDS presentation [61].

DDS can be thought of as one of the abovementioned complications and managed based on TCM syndrome differentiation. In other words, DDS can be categorized as TCM diseases such as “headache,” “dizziness syndrome,” “vomiting,” and “convulsion diseases” according to the clinical manifestations. Most modern TCM doctors believe that the cause of DDS is mainly exhaustion of the kidney and spleen causing the inability to distribute body fluid. For example, Guishun [64] proposed that DDS was a clinical manifestation of “Water Invasion disease.” Uremia patients usually have little urine or oliguria, which means that fluid overload cannot be expelled. To put it in another way, in the TCM viewpoint, blocking of the lower orifice and failure to send down turbid Yin lead to turbid Yin upwardly interacting with clear Yang, hence the syndrome of Water Invasion disease. Ho found that administration of modified Wu Ling San (WLS) decoction before HD could improve the response rate of DDS symptoms as compared to hyperosmotic fluid (100% versus 83.3%) by eliminating excess water in the brain. Li Pingduan [65] suggested that the pathogenesis of DDS could be both Yin and Yang exhaustion of both the kidney and spleen causing the inability to distribute inner fluids. They found modified Wen Yang Li Shui Tang (WYLST) to be effective for amelioration of DDS symptoms as compared with conventional therapy. The formula is composed of Ban Xia Bai Zhu Tian Ma Tang, WLS, Ze Xie Tang, Fu Ling Ze Xie Tang, and Zhen Wu Tang (ZWT), all of which address dysfunction of fluid metabolism in clinical TCM practice. Numerous researchers and systematic reviews have reported that the components of WYLST can strengthen the left ventricular ejection fraction, lower BP, and improve fluid retention [66–68]. However, the side effects of WYLST are similar to those of antihypertensive drugs, such as headache, a feeling of distension in the head, palpitation, drowsiness, and fatigue.

In addition to CHM, acupuncture can also be applied for DDS [69]. For instance, Taichong (LR3), Fengchi (GB20), Baihui (GV20), Sishencong (EXHN 1), Xingjian (LR2), Neiting (ST44), and Xiaxi (GB43) can be prescribed for dizziness. The Shao Yang Meridian acupoints can be stimulated to treat headache, for it is the meridian with the widest distribution over the head. In TCM theory, an ancient expression notes that “disorders of the abdomen can be treated by Zusanli (ST36).” ST36 belongs to the stomach meridian and has the effect of dispersing the meridian *Qi* of the stomach and spleen, balancing the *Qi* and blood, and harmonizing the Yin and Yang, so it is capable of antiemesis. However, research on acupuncture interventions for DDS is still sparse and has yet to develop persuasive evidence. Further large-scale, rigorous research will be necessary in the future.

## 8. Discussion and Future Perspective

HD is an important medical treatment for acute or chronic renal failure and other serious conditions. However, a variety of complications are common and can interrupt the HD to varying degrees, even reducing its quality. In addition, some complications are serious and even life-threatening. Here we provide a first review of TCM therapies, including body or auricular acupuncture, acupressure, HAT, moxibustion, and CHM, which are used for adjuvant management of HD-related complications. Diseases corresponding to TCM viewpoints, etiology and pathogenesis, and treatment options are summarized in Table 1. Because all of these therapies possess their own advantages and disadvantages, we cannot draw any conclusions on whether one method of adjuvant treatment is definitively superior to another, and little evidence is available to define optimal adjuvant management of such complications. In addition, the major symptoms and signs during HD, including hemodynamic change, electrolyte and fluid imbalance, and incomplete removal of uremic toxins, usually affect the individual constitution as both essence-*Qi* deficiency and waste accumulation in patients with ESRD, based on the theory of Yin-Yang [70]. Thus, a diverse methodology of tonifying and elimination are frequently applied in clinical practice.

**Figure 1:**
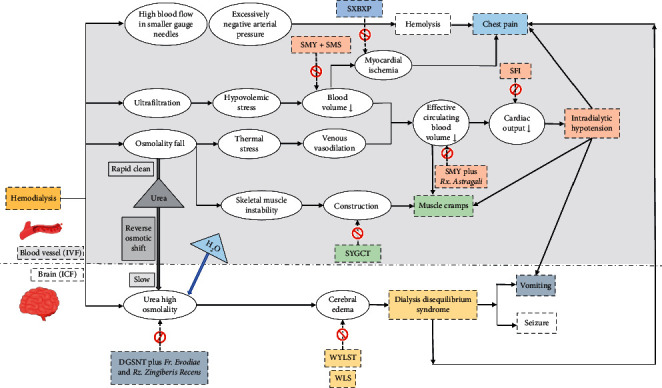
Application of Chinese herbal medicine formulas to hemodialysis-related complications: mechanisms and management (IVF: intravenous fluid; ICF: intracellular fluid).

Despite the promising effects of TCM in the treatment of HD-related complications, how CHM formulas work together and what their common targets are are still ambiguous. CHM formulas often use compound prescriptions involving various plants, minerals, and animal products, all comprising a variety of chemical constituents, which lead to a comprehensive response in the human body [71]. To clarify the mechanistic action of CHM formula for complex HD-related diseases from a system level, we proposed a mode to illustrate the mechanisms and management (Figure 1).

In our review article, we have found that few toxicity profiles of CHM formulas are available (Table 2). Perhaps the information provided in these references from China is insufficient to make a clear distinction. On the other hand, the information might verify that traditional scholars believe that CHM is relatively safe as long as the treatment is based on TCM syndromes. However, these traditional practices are still subjective and personal-experience-oriented, which can lead to diagnoses of the same patient by different TCM doctors being inconsistent or even contradictory. The toxicity of CHM formulas should be correctly recognized and reasonably utilized. Toad venom (*Venenum Bufonis*, Chan Su), for instance, is cardio- and neurotoxic when administered alone, but its toxicity may be reduced by extending the peak time of toad steroid ingredients with other compatible ingredients in SXBXP, thus demonstrating the scientific nature of compound compatibility [72]. Thus, it is a huge challenge to realize proper risk assessment of individual TCM products/herbs in TCM setting.

Not only is acupuncture effective for complications related to HD, such as uremic symptoms, insomnia, and fatigue, but also it is able to improve renal function, proteinuria, BP, anemia, and pain in CKD patients [73]. A single-blinded RCT found that acupuncture over Hegu (LI4), Zusanli (ST36), and Taixi (KI3) could improve creatinine levels and glomerular filtration rates after a treatment course of 12 weeks [74]. Moxibustion, a TCM prophylactic and curative treatment in which ignited moxa wool is applied over specific acupoints with the intention of warming the skin and stimulating circulation has also been found to have a therapeutic effect in a rat focal segmental glomerulosclerosis model. Shanghai University in China conducted a study to compare the efficacies of losartan and moxibustion [75]. FSGS rats were randomly divided into a model group, a losartan (positive control) group, a Shenshu (BL23) moxibustion group, and a Geshu (BL17) moxibustion group, and kidney function and renal pathological changes were monitored as the outcomes. After a 12-week intervention, urinary protein, serum creatinine, urea nitrogen, and serum uric acid were significantly reduced in the losartan and both moxibustion groups. In addition, renal *α*-SMA, FN, and TGF-*β* also decreased, while podocin and nephrin protein and mRNA increased. Hence, it could be concluded that moxibustion might significantly alleviate pathological damage in renal tissue, with efficacy similar to that of losartan.

Numerous articles have reported that integrating TCM with WM might have advantages over other methods of treating ESRD and may alleviate HD-related complications [10, 76, 77]. In one positive report, Kim et al. showed that acupuncture seems feasible and safe for symptom management in patients undergoing HD [76]. Case study data showed that Ren Shen Yang Rong Tang was more effective than WM in decreasing inflammatory markers and oxidative stress in HD at a 6-month follow-up [77]. For ESRD patients, persistent low-grade inflammation has been recently recognized as a risk factor for cardiovascular and all-cause mortality, as well as for the development of protein-energy wasting and other comorbid conditions [78]. Further investigations are needed to evaluate the effects of TCM targeting inflammation in ESRD. Scientific scrutiny of TCM is needed with regard to the safety, efficacy, and quality of TCM therapies; the accessibility to TCM agents; and the rational use of TCM. These objectives can be gradually achieved through public health policies and legislation.

## 9. Conclusion

As the dialytic population increases year by year, more and more patients will begin to receive HD treatment in the near future. To cope with the various clinical manifestations of HD complications, modern TCM doctors should treat the disease under the principle of the four diagnostics and the eight outlines of syndrome differentiation instead of being limited by modern disease terms. To achieve sufficient high-quality care, the efforts of the integrative medical team are only one component; the patient's understanding of the condition and compliance with the doctor's instructions, such as weight control during dialysis, electrolyte balance, dietary control, and pain care of the fistula, are also important.

TCM is simple, convenient, inexpensive, and effective, and it should be promoted to the general public. Currently, however, there is still not enough robust evidence for recommending TCM therapies. This review should serve only as an introduction, and we look forward to well-designed and methodologically rigorous studies being conducted in the future to confirm the efficacy and safety of TCM for HD complications and thereby benefit more HD patients.

## Figures and Tables

**Table 1:** Description of traditional herbal medicine viewpoints and treatments for acute hemodialysis-related complications in this review.

Complication	TCM disease	Symptom and sign	Etiology and pathogenesis		Method	Treatment	Reference
*Intradialytic hypotension*	Dizziness syndrome/juc collapse/asthenic disease	Cold clammy limbs, general weakness, shortness of breath, and sweating	Deficiency syndrome	Yang*-Qi* deficiency of spleen and kidney	NA	NA	[17]
			Excess syndrome	Blood stasis, dampness, windiness, and water vapor			
			Early stage	Deficiency of both *Qi* and Yin	CHM	Sheng Mai Yin, *Rx. Astragali*	[18]
			Late stage	*Qi* desertion along with fluid and blood depletion		Shen Fu decoction	
			Deficiency of kidney-Yang		HAT	*Semen Sinapis*, *Rhizoma Corydalis*, and *Hb. cum Rx. Asari* applied to Yongquan (KI1) and Guanyuan (RN4)	[19]
			Deficiency in origin and excess in superficiality	Dampness and heat with deficiency of both *Qi* and Yin	CHM	Sheng Mai Yin and Si Miao San decoction	[27]

*Intradialytic hypertension*	Dizziness syndrome	Headaches, fatigue or confusion, vision problems, chest pain	Hyperactivity of liver-Yang		Acu	Sanyinjiao (SP6), Taixi (KI3), and Shenguan (LE44)	[32]
			Deficiency of kidney-Yin		EA	Erbeixin (P1), Erjian (EX-HN6), liver (CO12), Jiaowoshang (TF1)	[33]
			Deficiency of Yin and Yang			Spleen (CO13), kidney (CO10), Erbeipi (P3), Erbeigou (PS)	
			Interior retention of phlegm and dampness			Sanjiao (CO17), spleen (CO13), Erbeipi (P3), Erbeigou (PS), Jiaogan (AH6a)	
			Deficiency of Yang floating upward		HAT	*Rz. Chuanxiong*, *Rx. Achyranthis Bidentatae*, *Fr. Evodiae* applied to Yongquan (KI1)	

*Muscle cramps*	Jin Bi/Bi-syndrome	Tonic contraction and myalgia	Stagnation of coldness in the muscles		Mox	Zusanli (ST36), Sanyinjiao (SP6), and Guanyuan (CV4)	[38]
			Malnutrition of liver, spleen, and kidney		Acupressure	Hegu (LI4), Taichong (LR3), Chengshan (BL57), Chengjin (BL56), Yanglingquan (GB34), and Zusanli (ST36)	[39]
						Chengshan (BL57), Shuigou (GV26), Taichong (LR3), Guanyuan (CV4), Qihai (CV6), Yongquan (KI1), Lieque (LU7), and Taiyuan (LU9)	[40]
			Deficiency of liver and kidney		Acu	Ququan (LR8), Fuliu (KI7), Guanyuan (CV4), Qimen (LR14), Ganshu (BL18), and Shenshu (Bl23)	[41]
			Deficiency of Yin and blood, malnutrition of the tendons and channels		CHM	Shao Yao Gan Cao Tang	[43, 44]

*Nausea and vomiting*	Retching/hiccup/vomiting	Nausea, vomiting, anorexia, allotriogeusia, headache	Deficiency of spleen and kidney		HAT	*Zingiber officinale* applied on Shenque (CV8)	[47]
					Acu	Zhongwan (CV12), Neiguan (PC6), and Zusanli (ST36)	
			Cold blocking the channels due to blood deficiency, incoordination between the spleen and liver resulting in rebellious *Qi*		CHM	Dang Gui Si Ni Tang plus *Fr. Evodiae* and *Rz. Zingiberis Recens*	[50]

*Headache*	True headache		Liver dysfunction in dispersion, deficiency of *Qi*, blood and Yin essence, and stasis of blood		EA	Shenmen (TF4), subcortex (AT4), occiput (AT3), kidney (CO10), liver (CO12), and spleen (CO13)	[53]

*Chest pain*	Chest impediment	Pressure, fullness, burning, or tightness in chest	Deficiency in origin and excess in superficiality	Deficiency of kidney-*Qi* and insufficiency of heart-blood, resulting in phlegm-dampness, or stasis blocking heart collaterals	CHM	She Xiang Bao Xin Pill	[56]

*Dialysis disequilibrium syndrome*	Headache/dizziness syndrome/vomiting/convulsion diseases	Nausea, headache, vomiting, restlessness, and even seizures and coma	Deficiency of spleen and kidney, insufficient gasification causes phlegm turbidity retention		CHM	Wu Ling San	[64]
					CHM	Wen Yang Li Shui Tang	[65]
					Acu	Taichong (LR3), Fengchi (GB20), Baihui (GV20), Sishencong (EXHN 1), Xingjian (LR2), Neiting (ST44), and Xiaxi (GB43)	[69]

Acu: acupuncture; CHM: Chinese herbal medicine; EA: ear acupuncture; HAT: herbal acupoint therapy; Mox: moxibustion; NA: not applicable.

**Table 2:** Summary of Chinese herbal medicine formulas: main effects, mechanisms, and possible toxicity profiles.

Name	Composition	Design	Dosage and route of administration	Main effect	Mechanism	Toxicity profiles
Shen Fu injection (SFI) [18]	*Rx. Ginseng, Rx. Aconiti Lateralis Preparata*	Case	20–30 ml, IVD	↑BP	Increment of heart rate and cardiac contractility	Aconite poisoning with arrhythmia and shock [18]

Sheng Mai Yin (SMY) and modified Si Miao San (SMS) decoction [27]	*Rx. Ginseng, Tub. Ophiopogonis,* and *Fr. Schisandrae, Sm. Coicis, Cx. Phellodendri, Rz. Atractylodis, Rx. Cyathulae*	Case control	A: SMY + SMS 200 ml, BID PO plus Sheng Mai injection (SMI) 30 ml, IVD 12 weeks; B: SMI 30 ml, IVD 12 weeks	↑BP, hemoglobin, and albumin ↓CRP	Elevation of BP and plasma colloid osmotic pressure, improvement of the microinflammatory state	Not reported

Shao Yao Gan Cao Tang (SYGCT) [43]	*Rx. Paeoniae Alba, Rx. Glycyrrhizae Preparata*	Case series and animal	6 GM, PO QD 4 weeks	↓Episodes of muscle cramp, ↑sural NCV, ↓contraction of skeletal muscles in rats	Direct inhibition of muscle contraction due to nerve stimulation	Mild hyperkalemia (5.5–6 mEq/L) [18] or pseudoaldosteronism in long-term use [44]
Shao Yao Gan Cao Tang (SYGCT) [44]		UCT	A: 2.5 g, PO once at home; B: 2.5 g, PO once before HD 4 weeks	↓Episodes and duration of muscle cramp, ↓amount of saline infusion, ↑VAS	Inhibitory effect on excessive muscle contraction and promotion of plasma refilling in muscle	

Dang Gui Si Ni Tang (DGSNT) plus *Fr. Evodiae and Rz. Zingiberis Recens* [50]	*Rx. Angelica Sinensis, Rx. Paeoniae Alba, Ram. Cinnamomi, Hb. cum Rx. Asari, Rx. Glycyrrhizae Preparata, Fr. Jujube, Caul. Akebiae, Fr. Evodiae, Rz. Zingiberis Recens*	Case	3-4 g, PO TID 14 days	↓N/V, BUN	Reduction of BUN level, balancing of the PH value of the GI tract	*Fr. Evodiae*-hepatotoxicity in rat [51] antiplatelet effect *in vivo* [52]

She Xiang Bao Xin Pill (SXBXP) [56]	*Moschus, Rx. Ginseng, Cx. Cinnamomi, Calculus bovis, Styrax, Venenum Bufonis, Borneolum*	RCT	A: SXBXP 2 pills, SL PRN; B: isosorbide dinitrate 10 mg, SL PRN; C: SXBXP 2 pills + isosorbide dinitrate 5 mg, SL PRN	↓Chest pain and associated symptoms, ↓BP and HR	Dilation of coronary artery, increment of myocardial contractility, and reduction of heart rate	Coagulation dysfunction [58]; toad venom has digoxin-like effects [59]

Wu Ling San (WLS) decoction [64]	*Rz. Alismatis, Poria, Scl. Polypori, Rz. Atractylodis Macrocephalae, Ram. Cinnamomi*	RCT	A: WLS 100–150 ml, BID, PO once before HD; B: 50% glucose 40–60 ml, IVD once before HD	↓DDS symptoms (headache, nausea, anxiety, blurred vision)	Improvement of cerebral edema and decrement of CSF pressure	Not reported

Wen Yang Li Shui tang (WYLST) [65]	*Rx. Aconiti Lateralis Preparata, Poria, Rz. Atractylodis Macrocephalae, Ram. Cinnamomi, Rz. Zingiberis, Rx. Paeoniae Alba, Sm. Lepidii, Fr. Jujube, Rx Pseudostellariae, Tub. Ophiopogonis, Fr. Schisandrae, Fr. Amomi, Rx. Astragali, Rhodobryum roseum, Rx. Glycyrrhizae Preparata*	RCT	A: 20 GM, PO once upon DDS attack; B: Conventional therapy upon DDS attack	↑Response rate of DDS symptoms (96% versus 80%)	Increment of plasma osmolality and simultaneous decrement of further osmotic shift to brain	Not reported

BID: twice daily; BP: blood pressure; BUN: blood urea nitrogen; CRP: C-reactive protein; CSF: cerebrospinal fluid; DDS: dialysis disequilibrium syndrome; EKG: electrocardiogram; EMG: electromyogram; GI: gastrointestinal; HR: heart rate; IDH: intradialytic hypotension; IVD: intravenous drip; NA: not applicable; NCV: nerve conduction velocities; N/V: nausea and vomiting; PH: potential of hydrogen; PO: oral; PRN: pro re nata; QD: once daily; RCT: randomized controlled trial; SL: sublingual; TID: three times daily; UCT, uncontrolled trial; VAS: visual analogue scale.
